# Association of Blood Urea Nitrogen with Cardiovascular Diseases and All-Cause Mortality in USA Adults: Results from NHANES 1999–2006

**DOI:** 10.3390/nu15020461

**Published:** 2023-01-16

**Authors:** Canlin Hong, Huiping Zhu, Xiaoding Zhou, Xiaobing Zhai, Shiyang Li, Wenzhi Ma, Keyang Liu, Kokoro Shirai, Haytham A. Sheerah, Jinhong Cao

**Affiliations:** 1 Department of Epidemiology and Biostatistics, School of Public Health, Wuhan University, Wuhan 430071, China; 2 School of Public Health, Capital Medical University, Beijing 100069, China; 3 Center for Artificial Intelligence Driven Drug Discovery, Faculty of Applied Sciences, Macao Polytechnic University, Macau 999078, China; 4 Public Health, Department of Social Medicine, Osaka University Graduate School of Medicine, 2-2 Yamadaoka, Suita-shi 565-0871, Japan; 5 International Collaborations Ministry of Health, Riyadh 11176, Saudi Arabia; 6 School of Management, Hubei University of Chinese Medicine, Wuhan 430065, China; 7 Research Center for the Development of Traditional Chinese Medicine, Hubei Province Project of Key Research Institute of Humanities and Social Sciences at Universities, Wuhan 430065, China

**Keywords:** blood urea nitrogen, cardiovascular diseases, all-cause mortality, National Health and Nutrition Examination Survey (NHANES)

## Abstract

In the general population, there is little evidence of a link between blood urea nitrogen (BUN) and long-term mortality. The goal of this study was to explore whether higher BUN concentration is a predictor of cardiovascular disease (CVD) and all-cause mortality. From 1999 to 2006, the National Health and Nutrition Examination Survey (NHANES) included 17,719 adult individuals. Death outcomes were ascertained by linkage to the database records through 31 December 2015. The Cox proportional hazard regression model was used to calculate hazard ratios (HRs) and 95% confidence intervals (CIs) for CVD and all-cause mortality in individuals. We also performed stratified analyses based on age, gender, drinking, smoking, history of hypertension and diabetes. During a mean follow-up 11.65 years, a total of 3628 deaths were documented, of which 859 were due to CVD. Participants with higher BUN had a higher risk of CVD and all-cause death compared to those with lower BUN. After multifactor adjustment for demographics, major lifestyle factors, and hypertension and diabetes history, higher BUN levels compared with lower levels were significantly associated with higher risk of CVD (HR: 1.48 [1.08, 2.02], *P*-trend < 0.001) and all-cause mortality (HR: 1.48 [1.28, 1.72], *P*-trend < 0.001). In subgroup analyses, we found that the trend in the association of BUN with the risk of death remained strong in female subjects. Greater BUN levels were linked to higher CVD and all-cause mortality in the NHANES of American adults. The importance of BUN in predicting death is supported by our research.

## 1. Introduction

Cardiovascular disease (CVD) is a primary cause of morbidity and death around the world [1,2]. The number of deaths from CVD has climbed by 12.5 percent globally in the last decade [3]. CVD is responsible for nearly a third of all fatalities worldwide [4]. Ischemic heart disease (IHD), stroke, hypertensive heart disease (which eventually leads to heart failure), cardiomyopathy, rheumatic heart disease (RHD), and atrial fibrillation account for about 95% of all CVD deaths [5]. The majority of CVD can be avoided by addressing behavioral risk factors, such as cigarette use, poor diet, obesity, physical inactivity and alcohol abuse. Therefore, it is critical to recognize CVD as soon as possible so that the prevention and control may begin with counseling and risk factors [6].

Blood urea nitrogen (BUN) is a protein metabolic waste produced by the liver and excreted by the kidneys [7], and it is used as a biomarker to assess renal function on a regular basis [8]. Renal function indexes, such as BUN, glomerular filtration rate, and creatinine, have been linked to CVD mortality in the studies [9,10]. BUN levels have been found to be high in patients with CVD in clinical investigations, making this biomarker a useful predictor of CVD prognosis compared to other renal function markers [11,12,13]. A greater BUN level predicted mortality with acute coronary syndrome, according to the prospective study in 9420 patients [14]. BUN, on the other hand, is a neurohumoral activity and renal function marker that can represent the pathophysiology of CVD. Protein intake, corticosteroids, gastrointestinal hemorrhage, and dehydration are a few of the factors that alter BUN levels. Furthermore, BUN can indicate the link between nutritional status, protein metabolism and renal function, and is a key marker for metabolic illnesses and other diseases [15,16,17]. Several studies indicated that BUN/creatinine ratio was a well-known predictor of adverse outcomes independently from both BUN and creatinine in CVD patients [11,13]. However, it remains controversial whether BUN has effects that are independent of other clinical and laboratory characteristics in terms of predicting CVD mortality. In the states of renal hypoperfusion from hypovolemia, renovascular disease, or reduced cardiac output, BUN may change independent of creatinine or glomerular filtration rate due to enhanced urea reabsorption under the effects of the sympathetic nervous and renin-angiotensin-aldosterone systems [14]. These correlates of BUN and cardiovascular risk require more evidence in the large-scale population study.

Most studies on the relationship between BUN and mortality are conducted in specific groups, such as hospitalized patients [12,13,14]. Since the studies on the association of BUN with CVD and all-cause mortality in the general population are rare, it remains a controversial topic, requiring further well-designed prospective cohort studies. Thus, the objective of the study was to determine the correlations of BUN concentrations with CVD and all-cause mortality in National Health and Nutrition Examination Survey (NHANES).

## 2. Materials and Methods

### 2.1. Study Population

The National Health and Nutrition Examination Survey (NHANES) is an ongoing, independent, nationally representative cross-sectional survey of non-institutionalized USA civilian populations conducted by the National Center for Health Statistics (NCHS) every 2 years, and the primary objective of the study is to assess the health and nutritional status of adults and children in USA. From the date of interview to the date of death or review (31 December 2015), the death and underlying cause of death were assessed by linking to the death record. More details about NHANES have been described elsewhere [18].

**Figure 1:**
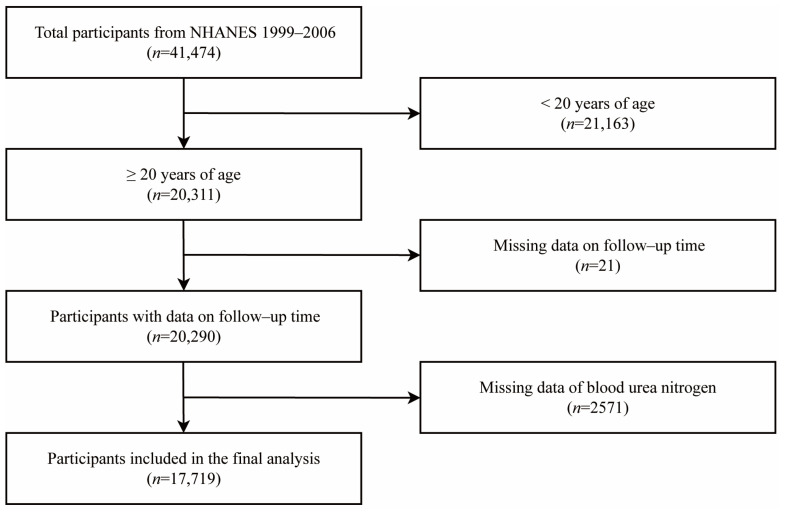
Flowchart of participants included in the analysis.

The population for this prospective cohort study was 41,474 USA subjects between 20 and 85 years old from the NHANES (1999–2006) with a mean 11.65-year follow-up. Participants were excluded if were younger than 20 (*n* = 21,163), had missing data on follow-up time (*n* = 21) or were lacking data of BUN (*n* = 2571). A total of 17,719 adult individuals were included in the final analysis (Figure 1). Data used in this study were derived from a de-identified and publicly database (https://www.cdc.gov/nchs/nhanes/index.htm (accessed on 11 May 2022)). The NHANES is approved by the National Center for Health Statistics Research Ethics Review Board, and all participants provided written consent.

### 2.2. Exposure Assessment and Confounding Factors

Samples of blood urea nitrogen were from non-hemolytic samples from fasted subjects. Separate serum or plasma were with EDTA, heparin, citrate, or fluoride anticoagulants from cells within 1 h of collection. Serum was stored in 2.0 mL Nalge tubes. Specimens stored longer than 24 h should be frozen at −20 °C. Specimen stability has been demonstrated for 1 year at −20 °C. The criteria for unacceptable specimens were low volume (<0.25 mL), hemolysis, improper labeling, and prolonged contact of serum or plasma with cells.

Anthropometric data were collected by trained health technicians in the mobile examination center. The following covariates were extracted for the analysis: age (20–40, 40–60, and ≥60 years), gender (male or female), race (non-Hispanic white, non-Hispanic black, Mexican Americans, and others), educational level (< high school, high school, and > high school), body mass index (BMI) (<25.0, 25.0–29.9, and ≥30.0 kg/m^2^), alcohol consumption (none, moderate, and heavy drinkers), smoking (none, former, and current smokers), history of hypertension (yes or no), and history of diabetes (yes or no) were used as categorical variables. Total cholesterol (mg/dL), alanine aminotransferase (U/L), total protein (g/dL), albumin (g/L), globulin (g/L), and high-density lipoprotein (mmol/L) as continuous variables. Alcohol drinking status was categorized as never drinker (0 g/day), moderate drinking (0.1 to 27.9 g/day for men and 0.1 to 13.9 g/day for women), and heavy drinking (≥28 g/day for men and ≥14 g/day for women). Smoking status was classified as never smoker, former smoker, and current smoker, using two questions: “Smoked at least 100 cigarettes in life” and “Do you now smoke cigarettes”. Hypertension was defined as a mean blood pressure exceeding 140/90 mmHg for systolic pressure and diastolic pressure, respectively. History of diabetes was obtained by self-reporting or being diagnosed previously. For more information on obtaining these covariates, visit the NHANES website (https://www.cdc.gov/nchs/nhanes/ (accessed on 11 May 2022)).

### 2.3. Mortality Ascertainment and Follow-Up

From 2003 through 31 December 2015, death information was based on linked data provided through the Centers for Disease Control and Prevention (https://www.cdc.gov/nchs/data-linkage/mortality-public.htm (accessed on 11 May 2022)). The all-cause mortality was defined based on the International Classification of Diseases, 10th revision (ICD-10), and was assessed by the National Death Index (National Center for Health Statistics). Among the 9 detailed categories of underlying and other causes of death provided in the data, heart disease and cerebrovascular disease were used to assess cardiovascular disease mortality in the study (ICD I00-I09, I11, I13, I20-I51, I60-I69) [19]. Meanwhile, the follow-up time of the study was calculated from the NHANES 1999–2006 examination date until the last known date alive or censored through 31 December 2015.

### 2.4. Statistical Analysis

Considering the complex, multistage, probability sampling design of the NHANES survey, the representative participants of the certain civilian, noninstitutionalized USA subgroup population were oversampled. Thus, we integrated sample weight which had been created in the NHANES analysis.

Descriptive statistics were used to analyze the characteristic and distribution of the population. Cox proportional hazards models were used to estimate the hazard ratio (HR) and 95% confidence interval (CI) to identify the association between BUN and all-cause mortality and CVD mortality. Model 1 was adjusted for age, Model 2 was further adjusted for sex, race/ethnicity, educational level, BMI, alcohol drinking and smoking status. Model 3 was further adjusted for total cholesterol, ALT, total protein, albumin, globulin, and HDL. Model 4 was then adjusted for history of hypertension (yes or no) and diabetes (yes or no) based on Model 3. The corresponding interactions of age, gender, BMI, smoking, alcohol consumption, hypertension, and diabetes history were tested in model 4. Stratification by age (<60 versus ≥60 years), sex (male versus female), BMI (<25 versus ≥25), current smoker (Yes or No), current drinker (Yes or No), history of hypertension (Yes or No) and history of diabetes (Yes or No) were conducted for the association between BUN concentration and CVD and all-cause mortality. All analyses were performed using the SAS statistical package (Version 9.4; SAS Inc., Cary, NC, USA) and GraphPad Prism 8 software. Two-sided *p* values < 0.05 was considered statistically significant.

## 3. Results

### 3.1. Population Characteristics

Cohort characteristics of study participants at baseline for the four BUN groups were presented in Table 1. From 1999 to 2006, 17, 719 adults underwent self-reported investigations and laboratory tests, confirming 3, 628 deaths. Of these adults, 47.8% were male and 50.6% were Mexican–American. Participants with higher BUN concentrations were composed of more people who were older, who were men, who were Mexican–American, who had higher BMI, who drank more, and who had history of hypertension and diabetes.

### 3.2. CVD and All-Cause Mortality

Of the 17,719 participants, 3628 died during the mean follow-up period of 11.65 years, 859 of whom died from CVD. Table 2 showed the relationship between different BUN concentrations and CVD and all-cause mortality. We found that a higher BUN concentration was related to a higher mortality of CVD and all-cause mortality. In the fully adjusted model 4, we found that the risk of CVD mortality was significantly increased in the population with higher BUN concentrations (HR: 1.48 [1.08, 2.02], *P*-trend < 0.001). In addition, a similar association was found between BUN concentrations and all-cause death (HR: 1.48 [1.28, 1.72], *P*-trend < 0.001).

### 3.3. Stratification Analyses

**Figure 2:**
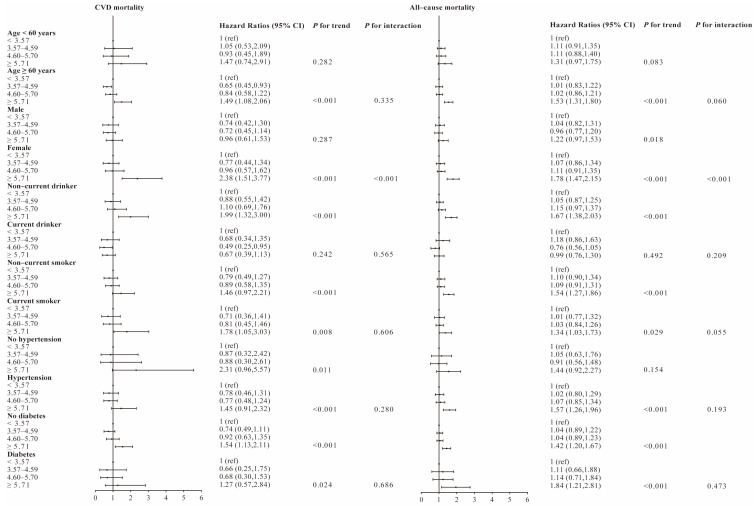
Associations between blood urea nitrogen and CVD, all-cause mortality stratified by age, gender, alcohol drinking status, smoking status, history of hypertension, history of diabetes. Hazard ratios were adjusted for age, sex, race/ethnicity, education, BMI, alcohol drinking status, smoking status, total cholesterol, ALT, total protein, albumin, globulin, HDL, history of hypertension and history of diabetes.

We conducted stratified analyses based on age, gender, alcohol drinking status, smoking status, history of hypertension and diabetes in Figure 2. The stratified analysis of six influencing factors was performed separately: age, sex, drinker, smoker, history of hypertension and diabetes. The results of the study showed that BUN levels in the Q4 group (≥5.71 mmol/L) were positively associated with all-cause and CVD mortality, especially in participants aged ≥ 60 years, female, non-current drinkers, current smokers, participants with history of hypertension and without history of diabetes. A negative correlation was found between only two groups of factors and CVD mortality: age ≥60 years Q2 group (3.57–4.59 mmol/L) and current drinker Q3 group (4.60–5.70 mmol/L). *P* for interaction is only significant in the sex hierarchy.

## 4. Discussion

In a nationally representative cohort of USA, BUN concentrations were associated with CVD and all-cause mortality. We also found a strong positive correlation between total BUN concentrations and CVD and all-cause mortality in participants aged ≥60 years, female, non-current drinkers, current smokers, participants with history of hypertension and without history of diabetes.

The results of this study were basically consistent with the previous studies on the relationship between BUN concentrations and CVD and all-cause mortality. BUN at admission and discharge was associated with both a 90-day mortality rate and a mortality rate in the first 36 months after admission [20]. Studies have shown that increased BUN at discharge is associated with increased all-cause mortality, which is 1.4–1.5 times higher risk than in patients without increased BUN [21]. Saygitov et al. also showed that an increase in serum urea nitrogen levels at the time of admission was associated with a more than fourfold risk of death [22]. Elevated BUN is associated with mortality and prognosis in many CVD patients. Patients with elevated BUN levels on admission are several times more likely to die from CVD events. BUN is a relatively common routine test that can effectively mark high-risk patients with acute coronary syndrome (ACS) for close monitoring of any adverse vascular events [23]. Increases in BUN and creatinine are highly prevalent in patients with ACS, with one in three patients having an increased level of either BUN or creatinine. Comparison of the prognostic significance of these factors showed that BUN is a more valuable predictor of ACS prognosis than creatinine [21].

Among other biomarkers associated with increased CVD risk (troponin-I, BNP, and CRP), the association between BUN and CVD outcomes remains significant, even a minimal increase in BUN may be a sign of adverse consequences [14]. Several studies have demonstrated that elevated BUN levels are associated with adverse outcomes in patients with heart failure (HF), especially in the setting of acute decompensation [24]. In a study of 541 patients with decompensated HF, Aronson compared the effects of serum creatinine, BUN, estimated creatinine clearance, and BUN/creatinine ratio on mortality. In the unadjusted analysis, all four indicators were significantly associated with all-cause mortality. However, after adjusting for other risk factors, this correlation was significant only for BUN and BUN/creatinine ratios, but not for serum creatinine or estimated creatinine clearance [25]. Moreover, it has been shown that in-patients admitted for episodes of HF decompensation, increase in BUN level during hospitalization, independent of admission level, is also a predictor of adverse outcomes after discharge [26]. The study showed that for in-patients with chronic stable HF, elevated BUN levels are still strongly associated with short and long-term mortality [27]. For in-patients with advanced heart failure, BUN is even better than N-terminal pro-brain natriuretic peptide in predicting mortality [28]. Compared with previous studies, this study mainly explored the association of BUN and CVD, all-cause mortality in the general population. The results of the study are more representative and may be more convincing.

In this study, stratified analysis showed a more significant association between BUN and CVD mortality in participants ≥60 years of age, women, current smokers, non-current drinkers and participants without history of diabetes. The association between BUN and all-cause mortality was more pronounced in participants ≥60 years of age, women, non-current drinkers, and participants with history of hypertension. When stratified by current smoking status or history of diabetes, there remain significant correlations between BUN and all-cause mortality in both different subgroups. A positive correlation between BUN and CVD, all-cause mortality was observed in participants ≥60 years of age. It is possible that the decrease in renal function is caused by a variety of factors due to the increase in age. These factors are also closely related to the cardiovascular system. These include changes in cardiac output, the use of angiotensin-converting enzyme inhibitors or diuretics, the use of contrast agents during vascular reconstruction, and the activation of the neurohormonal vasoconstriction system [29,30,31]. It is well known that smoking is associated with a high risk of acute myocardial infarction [32]. Additionally, smoking may increase the risk of renal dysfunction by enhancing renal inflammation, oxidative free radicals and fibrosis [33]. In a study of a Chinese male population, BUN levels were significantly lower in the smoking group than in the non-smoking group [34]. This is inconsistent with our stratification results because we included the whole population, whereas women had higher rates of muscle protein synthesis and higher rates of whole-body protein turnover, regardless of their muscle mass, BMI, and age [35,36]. This was also verified in our gender stratification. Current epidemiological studies attempting to link BUN to kidney disease are inconclusive, and there is little experimental evidence of a direct link between chronic alcohol consumption and kidney damage [37]. However, the association between alcohol consumption and cardiovascular disease is well established, with heavy alcohol consumption increasing the risk of alcoholic cardiomyopathy, hypertension, arrhythmias, and hemorrhagic strokes [38]. In addition, a recent large cohort of 1,337,452 USA veterans provided epidemiological evidence supporting the finding that higher BUN concentrations were associated with an increased risk of diabetes [39]. However, this result is inconsistent with our study. This may be related to the fact that diabetes is one of the most important risk factors for CVD, and its increased morbidity and mortality are due to vascular inflammation and endothelial dysfunction [40]. This makes no significant difference in mortality among people with diabetes.

The associations between higher BUN concentration and the risk of CVD, all-cause mortality may be related to the following mechanisms: renal dysfunction represents, in part, acute and predominantly hemodynamic renal function impairment. In the absence of urea-promoting agents, such as gastrointestinal bleeding, corticosteroid therapy, or a high-protein diet, the increase in BUN levels is often due to a decrease in glomerular filtration rate. Approximately 40% to 50% of the filtered urea is generally reabsorbed, mainly through the proximal tubules, where it is associated with the reabsorption of sodium and water. However, since urea reabsorption is a passive process associated with sodium and water reabsorption, it is driven by an increase in sodium content and reabsorption of water. Thus, increased sodium reabsorption in HF results in increased urea reabsorption [25]. A higher BUN level at baseline may suggest renal disease and a proven association with coronary artery disease [41]. On the other hand, BUN can not only reflect renal function, but also be related to the activation of neurohormones [42]. The increase in BUN reflects the cumulative effect of hemodynamic and neurohormonal changes, which leads to renal perfusion insufficiency [43]. Elevated vasopressin levels in HF may result in increased reabsorption of BUN. It may be related to atherosclerosis, as oxidative stress acts on the vascular wall, eventually leading to myocardial ischemia or infarction [44]. Similarly, the sympathetic nervous system and renal angiotensin system are upregulated in the onset of ACS, both of which are associated with increased tubular BUN reabsorption [45]. Furthermore, the effect of neurohormones is exacerbated by diuretic-induced depletion of the intravascular volume, which leads to renal hypoperfusion, for example, the activation of the renin–angiotensin–aldosterone system, increased catecholamine production and increased endothelin levels contribute to arteriole vasoconstriction [46].

Actually, there exist differences between BUN as a predictive variable and a causal variable. As a predictive variable, BUN can reflect lots of pathophysiological situations including hemodynamic and neurohormonal changes, which are related to poor CVD outcomes. These relationships between BUN and CVD mortality are supported by the large-scale population evidence in our study. However, as a causal variable, the causality between BUN and adverse outcomes of CVD still remains unclear. Renin–angiotensin–aldosterone system activation, renal sympathetic nervous system activation, oxidative stress and inflammation may serve as the potential underlying mechanisms which should be further explored in future clinical and epidemiological studies.

## 5. Strengths and Limitations

The main advantages of this study include the use of nationally representative sample data for long-term follow-up. To make the results more reliable, we weighed the hazard ratio. In addition, the analysis included adjustment for multiple confounders and stratified analyses to confirm robustness and heterogeneity of the results.

The study also has several limitations. First, since the information was self-reported, we cannot avoid possible measurement errors. Second, we were unable to assess the impact of changes in BUN during the CVD and all-cause mortality visits because NHANES 1999–2006 collected BUN information at baseline only, so it may be considered to repeat measurements in the future. The NHANES Associated Death Rate Profile 1999–2006 identified the cause of death by association with a national death index based on a death certificate. Although this method was validated by the Centers for Disease Control and Prevention (CDC) and is used in many CDC reports or related published reports, we cannot rule out the possibility of error in the classification of the cause of death. Finally, although we have adjusted for a wide range of confounders, there may be residual or unmeasured confounding.

## 6. Conclusions

Higher BUN concentrations are associated with higher risk of CVD and all-cause mortality. Reduced BUN concentration is beneficial to cardiovascular health and long-term survival.

## Figures and Tables

**Table 1:** Baseline demographic characteristics of the study population, according to quartiles of BUN.

Characteristic	Blood Urea Nitrogen (mmol/L)				*p* Value
	Q1 <3.57	Q2 3.57–4.59	Q3 4.60–5.70	Q4 ≥5.71	
**Number of participants**	4047	4661	4349	4662	
**Age, y**					<0.001
20–40	2400 (54.4)	2039 (46.5)	1315 (35.6)	655 (19.7)	
40–60	1107 (36.9)	1561 (39.4)	1462 (41.1)	1160 (36.6)	
≥60	540 (8.7)	1061 (14.1)	1572 (23.3)	2847 (43.7)	
**Gender**					<0.001
Men	1185 (31.8)	2115 (44.5)	2397 (55.3)	2769 (59.0)	
Women	2862 (68.2)	2546 (55.5)	1952 (44.7)	1893 (41.0)	
**Race/ethnicity**					<0.001
Non-Hispanic white	900 (8.8)	1152 (8.8)	997 (7.0)	878 (5.3)	
Non-Hispanic black	339 (9.5)	401 (11.6)	349 (10.1)	294 (8.0)	
Mexican American	1765 (65.4)	2102 (68.1)	2232 (74.2)	2863 (80.3)	
Other	1043 (16.3)	1006 (11.5)	771 (8.7)	627 (6.4)	
**Education**					<0.001
<High school	446 (6.0)	631 (6.4)	657 (6.2)	880 (9.0)	
High school	1766 (42.5)	1807 (35.6)	1676 (37.7)	1845 (39.0)	
>High school	1830 (51.5)	2215 (58.0)	2010 (56.1)	1924 (52.0)	
**BMI, kg/m^2^**					<0.001
<25.0 (Normal)	1432 (40.2)	1593 (36.2)	1341 (33.2)	1501 (32.3)	
25.0–29.9 (Overweight)	1273 (29.5)	1590 (32.8)	1581 (35.4)	1720 (36.3)	
≥30.0 (Obese)	1342 (30.3)	1478 (31.0)	1427 (31.4)	1441 (31.4)	
**Alcohol drinking status**					<0.001
Never drinker	2982 (72.8)	3293 (70.5)	2997 (68.9)	3377 (71.6)	
Moderate drinking	336 (8.6)	496 (11.9)	559 (13.8)	541 (13.2)	
Heavy drinking	532 (18.6)	694 (17.6)	609 (17.3)	531 (15.2)	
**Smoking status**					<0.001
Never smoker	2187 (48.7)	2443 (51.3)	2193 (49.6)	2313 (51.3)	
Former smoker	713 (16.7)	1021 (21.7)	1266 (28.4)	1686 (33.5)	
Current smoker	1143 (34.6)	1189 (27.0)	885 (22.0)	657 (15.2)	
**Total cholesterol (mg/dL)**	201.06 ± 46.07	201.39 ± 42.09	203.81 ± 41.45	203.38 ± 43.81	0.004
**ALT (U/L)**	22.98 ± 17.82	26.65 ± 23.01	27.08 ± 38.91	24.92 ± 31.52	<0.001
**Total protein (g/dL)**	7.16 ± 0.60	7.34 ± 0.50	7.35 ± 0.49	7.29 ± 0.51	<0.001
**Albumin (g/L)**	40.58 ± 4.86	42.87 ± 3.51	43.18 ± 3.20	42.47 ± 3.49	<0.001
**Globulin (g/L)**	31.03 ± 4.73	30.51 ± 4.58	30.33 ± 4.48	30.44 ± 4.74	<0.001
**HDL (mmol/L)**	1.47 ± 0.45	1.37 ± 0.41	1.35 ± 0.40	1.34 ± 0.41	<0.001
**History of hypertension**					<0.001
Yes	833 (20.0)	1184 (22.9)	1377 (27.7)	2173 (40.9)	
No	3180 (80.0)	3419 (77.1)	2943 (72.3)	2460 (59.1)	
**History of diabetes**					<0.001
Yes	216 (4.3)	314 (4.9)	387 (6.0)	827 (13.3)	
No	3794 (94.9)	4277 (93.9)	3892 (92.6)	3742 (85.2)	
Borderline	35 (0.8)	69 (1.2)	64 (1.4)	91 (1.5)	

Values are weighted mean ± SE for continuous variables or weighted % for categorical variables. BMI, body mass index; ALT, alanine aminotransferase; HDL, high-density lipoprotein.

**Table 2:** Association of the concentration of BUN with CVD and all-cause mortality in U.S. adults aged at least 20 years.

Characteristic	Blood Urea Nitrogen (mmol/L)				*P* for Trend
	Q1 <3.57	Q2 3.57–4.59	Q3 4.60–5.70	Q4 ≥5.71	
**CVD mortality**					
Deaths, No. (%)	82 (1.5)	118 (1.4)	168 (2.3)	491 (6.9)	<0.001
Deaths/person-years	511/47,792	799/56,451	1222/53,031	2930/49,173	
Mortality/per 1000 person	15	14	23	69	
Unadjusted	1 [Reference]	0.93 (0.63,1.39)	1.52 (1.08,2.13)	5.01 (3.80,6.59)	<0.001
Model 1	1 [Reference]	0.66 (0.44,0.99)	0.74 (0.52,1.06)	1.54 (1.13,2.09)	<0.001
Model 2	1 [Reference]	0.69 (0.46,1.04)	0.78 (0.54,1.12)	1.57 (1.13,2.16)	<0.001
Model 3	1 [Reference]	0.77 (0.51,1.15)	0.87 (0.61,1.24)	1.65 (1.20,2.28)	<0.001
Model 4	1 [Reference]	0.76 (0.52,1.11)	0.85 (0.61,1.20)	1.48 (1.08,2.02)	<0.001
**All-cause mortality**					
Deaths, No. (%)	406 (7.5)	609 (9.2)	809 (12.6)	1804 (27.0)	<0.001
Deaths/person-years	2743/47,792	4458/56,451	6158/53,031	11,928/49,173	
Mortality/per 1000 person	75	92	126	270	
Unadjusted	1 [Reference]	1.18 (1.01,1.37)	1.58 (1.36,1.83)	3.79 (3.23,4.44)	<0.001
Model 1	1 [Reference]	0.89 (0.76,1.03)	0.85 (0.72,1.00)	1.34 (1.15,1.58)	<0.001
Model 2	1 [Reference]	0.96 (0.84,1.10)	0.95 (0.82,1.10)	1.47 (1.27,1.71)	<0.001
Model 3	1 [Reference]	1.07 (0.94,1.23)	1.07 (0.92,1.23)	1.59 (1.37,1.84)	<0.001
Model 4	1 [Reference]	1.06 (0.92,1.21)	1.05 (0.91,1.22)	1.48 (1.28,1.72)	<0.001

Values are n or weighted hazard ratio (95% confidence interval). Model 1: adjusted for age. Model 2: Model 1 + gender, race/ethnicity, education, BMI, alcohol drinking status, smoking status. Model 3: Model 2 + total cholesterol, ALT, total protein, albumin, globulin, HDL. Model 4: Model 3 + history of hypertension, history of diabetes. CVD, cardiovascular disease; BMI, body mass index; ALT, alanine transaminase; HDL, high-density lipoprotein.

## Data Availability

The datasets generated and analyzed during the current study are available in the National Health and Nutrition Examination Survey (NHANES), https://www.cdc.gov/nchs/nhanes/ (accessed on 11 May 2022).
